# Dental Surgery and Organic Heart Disease

**Published:** 1920-08

**Authors:** P. J. Calvy

**Affiliations:** Fond Du Lac, Wis


### DENTAL SURGERY AND ORGANIC HEART DISEASE

BY P. J. CALVY, M.D., FOND DU LAC, WIS

In discussing this subject I do not intend to detract in the least from the brilliant results attained by dental surgery in the treatment of disease caused by focal infection about the teeth; rather, I mean to call attention to the very real danger which at times and under special conditions exists when radical treatment by extraction is attempted. Special reference is made to organic disease of the heart.

In a given case in which focal infection does exist about the teeth, together with organic cardiac disease, it is fair to assume, other causes being excluded, that the condition is caused or aggravated by the infection, and treatment for its removal should be instituted; but before this is done, certain results are to be anticipated. Are we going to stir up a latent infection in the heart and create an acute condition which may endanger the patient's life? This is possible. It has come to be accepted as proof of the accuracy of the diagnosis and the necessity of the operation that if the condition for which relief is sought by extraction of teeth is temporarily aggravated after the operation, the cause of the trouble has been determined and the procedure was justified. The deduction is logical. It is common experience to note that an arthritis or neuritis becomes acutely painful after the infection which causes it is, so to speak, stirred up. It is called a reaction, and is seldom thought of in connection with cardiac disease; but when this reaction takes place and affects a vital organ, such as the heart, results may be serious. Here the diagnostician in internal medicine should take a stand and be the judge when it comes to determining methods of procedure; and dental surgery will have to develop a technic which will reduce this danger to a minimum, as it is positively to be reckoned with.

Drainage of the infected area, such as an apical abscess, is the result which is desired; but this result, at times, and under certain conditions, is precisely what is not obtained, unless proper technic and after-treatment are employed.

After extraction, if the tooth socket is examined at any time during the first three or four days, it is found to be filled with a firm clot of blood, which precludes drainage and affords an ideal soil for the rapid growth of bacteria; and the traumatism to the structures around the tooth greatly increases the opportunity for absorption. It is under such conditions that there is a rapid rise of temperature, increased pulse rate, and an acute attack or exacerbation of a chronic trouble, as exemplified by the history of the subjoined cases.

### REPORT OF CASES

CASE 1.—A thin, anemic woman, aged 42, came seeking relief for attacks of vertigo and general weakness, complaining also of having precordial pain and irregular heart action. Examination of the heart revealed a systolic mitral murmur, slight dilatation and an intermittent action, with nothing in the general history to account for the condition. The throat was negative. The teeth showed evidence of trouble. A hard swelling was found on the alveolar process of the jaw in the region of an impacted and partially unerupted third lower left molar. Pus was oozing out of a small opening on the top. The patient said that this condition had been present for five or six years. Roentgenograms of all the teeth revealed abscesses of the third lower left molar and second bicuspid. Extraction was advised, and the teeth were removed at the hospital the following day by a very competent man. Twelve hours later the temperature had risen to 102.5, and the pulse was 110; thirty-six hours later the temperature was 104, and the pulse 130. The tooth sockets were cleaned out and irrigated with an antiseptic solution, and in three hours the temperature had dropped to 100 and the pulse to 110. Twenty-four hours later another rise of temperature and pulse rate took place. Irrigation of the tooth sockets was again resorted to, with the same results. Thereafter irrigation and cleansing by antiseptic solutions prevented a further rise in temperature and increase in pulse rate.

On the fifth day after extraction, the heart trouble had become worse, the pulse rate remained high, the intermittent heart action was more pronounced, the murmur was more audible and the patient was very weak, being obliged to remain in bed for two weeks under treatment directed to the condition of her heart.

Case 2.—A woman, aged 64, had an exploratory laparotomy performed for suspected recurrent carcinoma in the pelvis, hysterectomy having been performed three years previously for cancer of the uterus. Examination before operation disclosed hypertrophy of the heart, the systolic blood pressure being 140, and the diastolic 110. A slight systolic murmur was present at the apex. The patient remained in bed in the hospital for a week before the operation. Laparotomy was performed, but it amounted to nothing more than an exploration, and I do not think it could be held responsible for the results, as the patient recovered from the short ether anesthetic and operation in a few days. On the eighth day after the operation she requested to have two lower second bicuspids extracted, saying that they were sore and had been giving her trouble for a long time. Extraction was done under local anesthesia. Twenty-four hours after extraction of the teeth, the temperature rose to 101, and the pulse to 125. Local treatment, as in the previous case, improved the symptoms somewhat. During the next twenty-four hours the pulse became weaker and more irregular, but the temperature did not rise above 100. The mitral murmur became more audible, the systolic blood pressure began to fall, the pulse rate and general weakness increased, and the patient died of dilatation of the heart at the end of the third day.

### COMMENT

Other and as typical cases can be reviewed from private practice and from the records of St. Agnes Hospital, where severe cardiac actions have occurred after the extraction of infected teeth.

It is not to be presumed that all patients with cardiac diseases are necessarily poor risks for extraction; but when the focus of infection to be eradicated is the probable cause of the heart trouble, the possibility of making matters worse must be borne in mind. No doubt valvular disease in well-compensated hearts, especially in young persons, can not be considered dangerous; but in older persons in whom the myocardium is degenerated, accompanied by valvular disease, when the energy index is low and cardiac decompensation is imminent, such hearts show that they are beginning to break under their load, and in such cases it behooves us to make haste slowly.—*Journal A. M. A.*, May 1, 1920.

